# Glaucoma treatment and deprivation: time-series analysis of general practice prescribing in England

**DOI:** 10.3399/BJGPO.2024.0005

**Published:** 2024-10-02

**Authors:** Jeremy Hooper, Cecilia Helen Fenerty, James Roach, Robert Anthony Harper

**Affiliations:** 1 Conclusio, Ty Derw Lime Tree Court, Cardiff Gate Business Park, Cardiff, UK; 2 Manchester Royal Eye Hospital, Manchester University NHS Foundation Trust, Manchester, UK; 3 Division of Pharmacy and Optometry, School of Health Sciences, Faculty of Biology, Medicine and Health, University of Manchester, Manchester, UK; 4 Division of Evolution and Genomic Sciences, School of Health Sciences, Faculty of Biology, Medicines and Health, University of Manchester, Manchester, UK; 5 Manchester Academic Health Sciences Centre, Manchester University NHS Foundation Trust, Manchester, UK

**Keywords:** drug prescribing, glaucoma, primary care

## Abstract

**Background:**

Despite advances in glaucoma management, topical eyedrop treatment has been paramount, with prostaglandin analogues (PGAs) being first-line agents. While late presentation is linked with deprivation, there is no recent research examining associations between deprivation and prescribing within primary care.

**Aim:**

To explore PGA prescribing in general practice over a 6-year timeline, assessing associations with deprivation.

**Design & setting:**

Analysis of NHS Business Services Authority (NHSBSA) data for general practice prescribing in England from April 2016–March 2022.

**Method:**

Glaucoma treatments by GP prescribers were extracted, identifying ~9.11–9.58 million prescriptions/annum. Data were linked to Index of Multiple Deprivation (IMD) quintiles of GP practices. Crude rates per 1000 population were calculated using population data from NHS Digital. Time-series analyses facilitated comparison in prescribing nationally and in deprived areas. Autoregressive Integrated Moving Average (ARIMA) modelling facilitated measurement of synchrony between time series using cross correlation.

**Results:**

PGAs and fixed combination eyedrops accounted for approximately two-thirds of glaucoma-related prescribing. Prescriptions per month increased slightly over a 6-year timeline, but rates per 1000 population reduced in 2020–2021 during the COVID-19 pandemic. The number of PGA prescriptions dispensed in deprived areas was lower than all other quintiles. Cross-correlation analysis indicates a lag of ~12 months between average PGA prescribing nationally versus more deprived areas.

**Conclusion:**

The rate of PGA prescribing in primary care was substantially lower in deprived versus affluent areas, with delayed uptake of PGAs in more deprived areas of ~12 months. Further research is needed to explore reasons for this discrepancy, permitting strategies to be developed to reduce unwarranted variation.

## How this fits in

Glaucoma is a leading cause of avoidable sight loss where prostaglandin analogue (PGA) eyedrops have traditionally been first-line treatment, formally embodied in recommendations within the National Institute for Health and Care Excellence (NICE) glaucoma guideline when first updated in 2017. Deprivation is linked to late presentation in glaucoma and has also been associated with a reduced likelihood of being treated with glaucoma medications, although no recent study, to the authors’ knowledge, has evaluated associations of glaucoma prescribing with socioeconomic status. PGAs and related fixed combinations accounted for approximately two-thirds of glaucoma-related prescribing, with prescriptions per month increasing slightly over the 6-year timeline 2016–2022, while prescribing rates per 1000 population reduced in 2020–2021 during the COVID-19 pandemic. The rate of PGA prescribing in general practice was substantially lower in deprived areas versus more affluent areas, and there was a delayed uptake of PGAs in more deprived areas, which requires further research to develop strategies to reduce inequality.

## Introduction

Glaucoma remains the second largest cause of sight loss in the UK, with a prevalence of ~4% in those aged >50 years,^1^ and with glaucoma care accounting for ~20% of hospital eye service workload.^2^ The Royal College of Ophthalmologists’ Way Forward project predicted the UK glaucoma population would increase by 22% from 2015 to 2025 and 44% from 2015 to 2035, conceding projections might underestimate demand if improved detection resulted in more prevalent cases converting to diagnosed cases requiring treatment.^3^

The purpose of glaucoma treatment is to slow ganglion cell loss and preserve patients’ vision and quality of life. Only lowering intraocular pressure has proven effectiveness in slowing progression.^4^ While surgery and selective laser trabeculoplasty (SLT) are important when appropriate, with the latter being recommended as first-line treatment within the updated NICE guideline in 2022,^5^ medical treatment has been the mainstay of treatment.^6^ Once diagnosed, prescribing typically remains in primary care supported by secondary care. Interestingly, while the first NICE guideline^7^ introduced recommendations for managing ocular hypertension (OHT) and chronic open angle glaucoma, this guideline did not change prescribing per se, potentially owing to recommendations embodying pre-existing practices.^8^ Indeed, PGAs were established as first-line treatment by 2003,^9^ and the impact of NICE updates^10^ in 2017 and 2022 have not yet been established. A recent Australian study^6^ observed prescribing rates remaining stable from 2001 to 2017, with PGAs being the most prescribed class. The expected hierarchy is PGA eye drops first line, beta blockers second line, with carbonic anhydrase inhibitors and alpha-agonists third-line choices.^11^

In relation to socioeconomic status (SES) and prescribing, one early study demonstrated those from more deprived areas were 8% less likely to be prescribed topical treatment for their glaucoma than those in more affluent areas.^9^ Heng *et al* ^12^ looked at geographical variations in glaucoma prescribing in England from 2008 to 2012. Using the Index of Multiple Deprivation (IMD), they found the upward trend of prescribing glaucoma medications was negatively associated with IMD. There is, however, a paucity of recent information on how SES is associated with glaucoma prescribing and whether earlier findings have changed during evolving NICE guidance. In this article, we report on glaucoma prescribing in general practice over ~6 years in England, focusing on PGA eyedrops and associations with SES.

## Method

Using prescribing data published by NHS Business Services Authority (NHSBSA)^13^ from April 2016 to March 2022, we extracted data relating to glaucoma treatment by GPs using British National Formulary codes, reflecting ~9.11–9.58 million prescriptions per annum. These data, for both PGA monotherapy and PGA-combination eyedrops, were linked to IMD quintiles of GP practices in each year. The Office for Health Improvement and Disparities calculates a deprivation score for each GP practice in England using IMD,^14^ used here to place practices in quintiles, where quintile one is least and quintile five the most deprived. We also used the GP registered population published by NHS Digital,^15^ allowing calculation of crude rates per 1000 population.

To compare the national trend in prescribing with that in more deprived areas, we undertook time-series analysis within Python to calculate moving averages, plotted against monthly data to ensure a good fit. We used 'statsmodel' (version 0.14.1) to fit an Autoregressive Integrated Moving Average (ARIMA) model to both time series, a method appropriate for understanding time series, which accounted for seasonality within data, with the autoregressive component recognising current values were based on historic data, while the moving average component assumed regression errors were linear. ARIMA models for overall national and most deprived data allowed measurement of synchrony between these time series using cross-correlation. The time series cross-correlation permitted understanding similarity of data in both time series and any delay in prescribing, which simple correlation would not permit, since it would compare correlation at the same time point.

## Results

### National analysis

**Figure 1.**
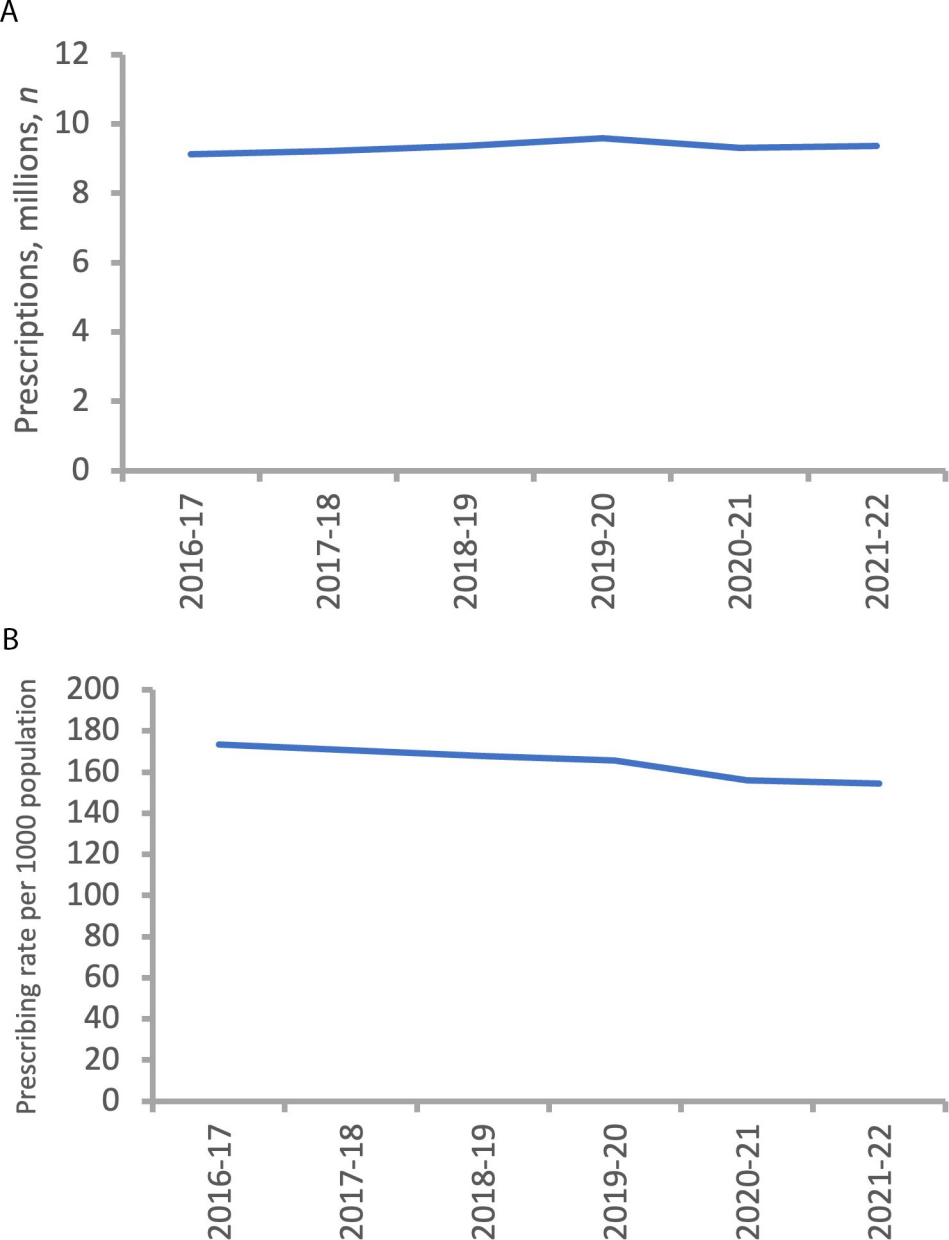
Trend in the number (**A**) and the rate (**B**) of glaucoma-related prescriptions in England (2016–2022). Numbers from NHS Business Services Authority;^12^ population data from NHS Digital.^13^

The annual trend for number and rate of glaucoma-related prescriptions in England is shown in Figure 1, ranging from a monthly average of 759 592 prescriptions in 2016–2017 to 798 482 prescriptions in 2019–2020 (~9.11–9.58 million prescriptions/annum). The number of prescriptions per month increased slightly over 6 years (Figure 1A). Over the period, the overall population grew by 15% (quintile range 12%–19%), while overall prescribing grew by 9% (quintile range 8%–11%). As the growth in population was greater than the growth in prescribing rate, Figure 1B shows a downward trend. The 95% confidence intervals (CIs) are excluded from Figure 1 for clarity but are very small owing to large numerators and/or denominators. For example, the national trend for the 2021–2022 prescribing rate was 154.38 (95% CI = 154.28 to 154.47).

**Figure 2.**
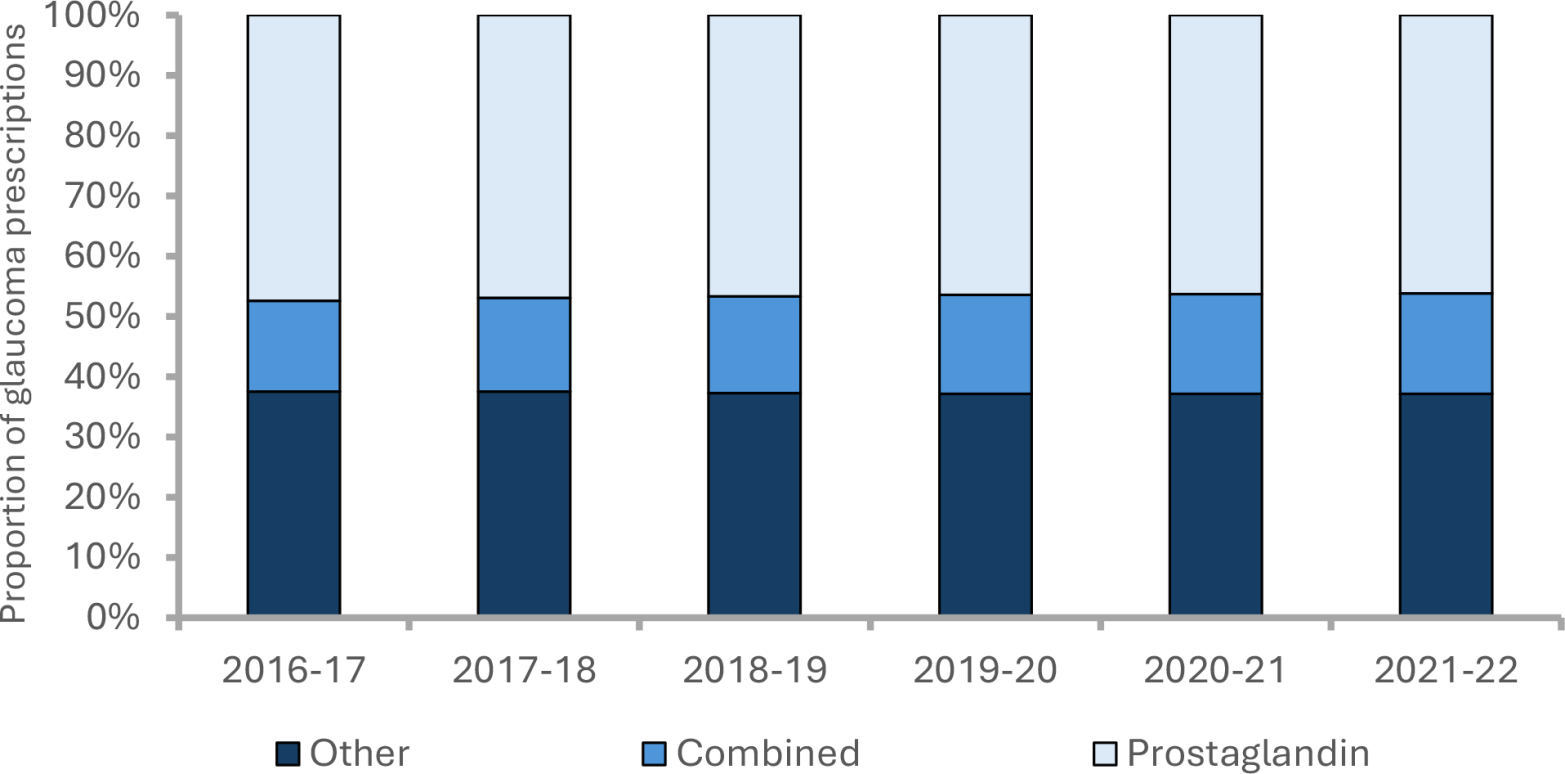
Trend in the proportion of glaucoma prescriptions by grouping (PGA, PGA fixed combinations, and ‘other’) in England (2016–2022). Data from NHS Business Services Authority.^12^ PGA = prostaglandin analogue.

We categorised glaucoma medications as follows: PGAs; fixed combination PGA eyedrops; and ‘other’ medications. Figure 2 shows the annual trend in these groups, with PGAs and related combinations accounting for two-thirds of prescribing, proportions changing little over time. Given NICE recommendations and PGA dominance, our remaining analyses focuses on potential associations with SES examining these medicines.

### Analysis by deprivation quintile

**Figure 3.**
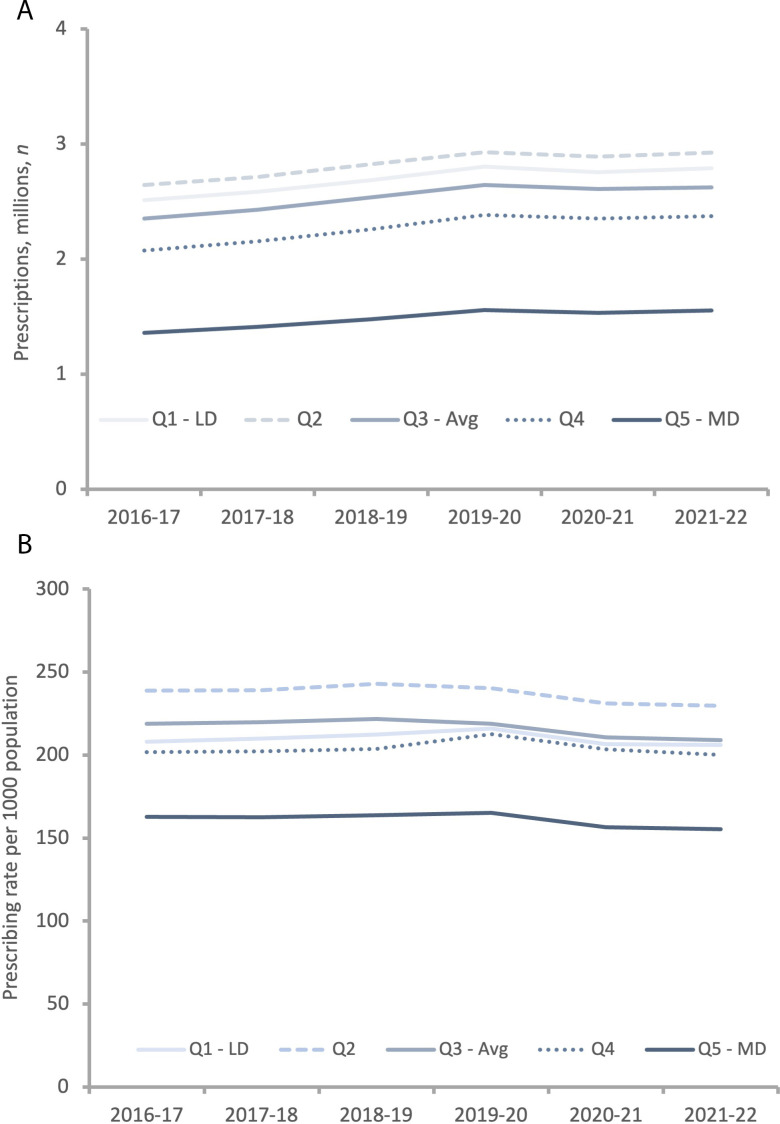
Trend in number (**A**) and adjusted rate per 1000 population (**B**) of glaucoma-related prescriptions by deprivation quintile in England (2016–2022). Numbers from NHS Business Services Authority;^12^ population data from NHS Digital.^13^ Avg = average. LD = least deprived. MD = most deprived.

Figure 3 shows prescribing over the last 6 years. Figure 3A demonstrates the number of prescriptions dispensed in the most deprived areas was lower than in other quintiles. Furthermore, disparity in prescriptions dispensed between most and least deprived increases slightly over time. The highest number of prescriptions dispensed occured in quintile 2. Figure 3B illustrates the prescribing rate per 1000 population, demonstrating a slight decrease across all quintiles. The greatest decline in prescribing occurred during 2019–2020 to 2020–2021, coinciding with the COVID-19 pandemic. There is a clear disparity in prescribing between the fifth and other quintiles; however, the difference between the quintile with the highest (quintile two) versus the lowest (quintile five) prescribing rate remained relatively constant, with 75 fewer people per 1000 population in quintile five being prescribed PGAs. Rate of prescribing PGAs in quintiles one, three, and four became more equitable over time, with a difference of 16 per 1000 population in 2016–2017 reducing to 9 per 1000 population by 2021–2022, resulting in little disparity between groups; however, the gap widened between the two most deprived quintiles (four and five), with the difference in prescribing increasing from 39 per 1000 population in 2016–2017 to ~45 per 1000 population in 2021–2022.

### Time-series analysis

Time-series analysis allowed us to understand the trend in the uptake of PGAs in primary care and, in particular, if the rate of uptake was slower in more deprived areas compared to the England average. The chosen analysis has three parts. Analysis of stationarity using the augmented Dickey–Fuller test (null hypothesis of non-stationarity and an alternative hypothesis of stationarity) showed the monthly trend data were stationary (*P* = 0.33 for national data and *P* = 0.22 for most deprived area data). Since these data were stationary, we fitted the ARIMA model with the 12-month rolling moving average data, removing random fluctuations in prescribing over the days in the month. The national moving average rose from ~475 000 to just over 500 000 (an increase of ~6%), while in the most deprived quintile the moving average rose from ~70 000 to just over 80 000 (a rise of ~14%). However, in the last months of the series, the trend was similar (data not shown).

**Figure 4.**
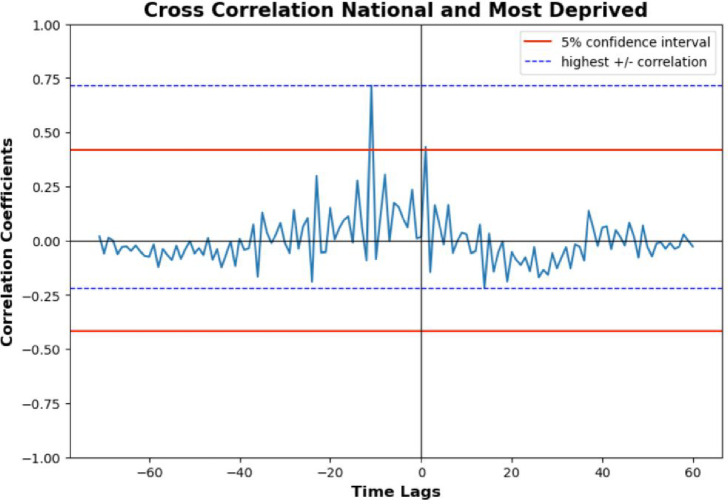
Cross-correlation analysis for national and most deprived area prescribing time series. Data from NHS Business Services Authority.^12^

Using ARIMA modelling allowed us to undertake further analyses. Figure 4 shows cross-correlation analysis, permitting visualisation of whether there is a lag between these two time series over time (that for overall national versus that for the most deprived data), rather than using simple correlation that returns a single value at a point in time. Interestingly, national data were held in place and created both lags and leads for data from the most deprived area, providing a more holistic view of the relationship between the time series. The highest positive correlation coefficient of ~0.7 occured at a time lag -12, suggesting a relatively strong positive correlation between prescribing in more deprived areas ~12 months after national prescribing. The peak correlation coefficient being outside the 95% CI suggests this correlation is unlikely to have occurred by random chance alone. Based on this analysis, there appears to be a lag of ~12 months between average prescribing in the more deprived areas versus prescribing nationally.

## Discussion

### Summary

Our study demonstrates PGAs accounted for approximately two-thirds of general practice prescriptions for glaucoma-related diagnoses in England over 6 years. After the first NICE guideline update in 2017, use of PGAs increased for the next 3 years to ~640 000 prescriptions per month, a ~10% increase. When analysing prescribing per 1000 population, there was an overall downward trend over the 6-year period, a finding potentially contrasting to the expected increase in at-risk cases needing treatment. In April 2020, there was a more rapid prescribing decline as COVID-19 impacted on sight testing, with subsequently reduced referrals and fewer new diagnoses. Significantly, in more deprived areas, crude prescribing per 1000 population was substantially lower than in more affluent areas, suggesting matters have not improved since Owen *et al*’s early study.^9^ Factors influencing this finding may include lower referral from primary care optometry within areas of deprivation, more advanced presenting glaucoma within deprived areas (leading to surgery as part of primary treatment or escalation to surgery after initial PGA prescribing), and poorer medication adherence. The latter may be linked to inadequate patient education and/or language provision, impacting on attendance and culminating in failure to refill prescriptions.

### Strengths and limitations

A strength of this work is collation of large-scale glaucoma prescribing in the NHS in England. Arguably these data (>9 million prescriptions per annum) reflect an unbiased source. However, while these data provide a complete picture of primary care prescribing in England, secondary care prescribing data, even when community dispensed, were not captured. Further, the dataset was not a record level dataset, meaning it did not include demographic information, for example, age or ethnicity. In addition, the analysis relies on the GP practice base for deprivation, rather than providing a view of deprivation for individuals receiving prescriptions. The IMD score was updated in 2019 and deprivation scores are relatively up-to-date descriptors of deprivation status. We acknowledge that this 2019 version may be outdated. Furthermore, allocation of one IMD score per practice may not adequately reflect populations, which likely has patients with different IMD levels, an acknowledgement of the well-reported complexity of SES, neighbourhood deprivation, and health.^16^ However, despite limitations, IMD remains the best readily available method for examining deprivation, while accepting some areas may have seen improvements in SES, for example, 'gentrification' masking existing population requirements, causing apparent improvement in deprived areas.

A further limitation is that our analyses were restricted to PGA prescribing, and future analyses may usefully include all glaucoma medications. Furthermore, we concede the historic nature of the data. We were not able to identify individuals, duration of using PGAs, or glaucoma stage at diagnosis. People presenting with advanced glaucoma may be managed differently, with NICE recommending early surgery, hence overall PGA prescribing may be lower owing to surgical versus medical management; however, this explanation was not borne out by a study of SES in cases undergoing trabeculectomy,^17^ where drainage surgery was carried out less frequently in patients from areas of greatest deprivation. While it is uncertain whether surgical management impacts PGA prescribing in areas of deprivation, there is evidence patient adherence may be influential. A study looking at demographics of patients registered severely sight impaired from glaucoma^18^ found those with advanced disease and those with poor adherence were more likely to have greater deprivation. Arguably, the corollary is increased sight testing in affluent areas generates more glaucoma referrals and greater repeat prescribing within primary care. Further research is necessary to better understand these differences, although since initial prescribing is largely in secondary care (covering populations from the most and least deprived areas), with repeat primary care prescribing supported from secondary care, it seems likely differences in deprived area versus national data include fewer referrals, more advanced presenting disease, and prescription refill factors.

### Comparison with existing literature

The majority of glaucoma cases are detected through case finding in primary care optometry. Day *et al* ^19^ showed a mismatch between areas of deprivation and location of optometrists, a finding supported by our own recent analysis,^20^ with both studies supporting the view that the optometry business model may deter practice establishment within deprived communities,^21^ creating a barrier to sight testing, impacting on detection. Several studies have looked at access to eyecare and SES. Knight *et al*’s review^22^ noted seven of eight high-quality studies concluded there *was* a significant positive association between lower SES and glaucoma stage at presentation, and a significant negative association between SES and secondary care attendance. An early case-control study examining deprivation and stage of presentation^23^ found deprivation was associated with late presentation, an important risk factor for subsequent blindness. A cohort study in Manchester investigating SES and vision loss in glaucoma^24^ found patients from deprived areas presented with more advanced loss, while SES has been shown as a risk factor for patients with acute primary angle closure.^25^ More recently, the relationship between late presentation of glaucoma and deprivation has been revisited, with Rathore *et al* ^26^ confirming the association between IMD and advanced visual field loss at diagnosis, while concluding rapid worsening of glaucoma during follow-up was not associated with IMD, suggesting equity of care and outcomes once patients were referred into the English hospital eye service. This latter suggestion is supported by King *et al*,^27^ who observed in their treatment of advanced glaucoma study that while SES at baseline is correlated with poorer vision it did not impact on the success of treatment at 24 months.

Some previous studies have described associations between primary care prescribing and deprivation in general medical prescribing. A recent study by Mooney *et al* ^28^ found drug categories most strongly correlated with deprivation included analgesics. Conversely, both hormone replacement therapy and combined oral contraception were prescribed more in affluent areas. Ophthalmic drugs did not feature in reporting of stronger associations between either deprivation or affluency, although interestingly Latanoprost was weakly positively correlated with deprivation. Overall, Mooney *et al* found SES correlated with *higher* rates of prescriptions for a large number of drugs, with only a few drugs being correlated with affluency. For glaucoma and our study, it is pertinent to note that in contrast to conditions noted in Mooney’s study, patients with glaucoma typically remain asymptomatic, potentially even where disease is advanced but asymmetric, and care-seeking behaviour before presentation may influence prescribing in a scenario different to the management of chronic pain, for example.

In relation to the downward trend in prescribing during COVID-19, contemporaneously reduced activity in secondary care resulted in backlogs, arguably a scenario influencing patients’ behaviours around continuation with glaucoma medications, owing to follow-ups being cancelled or delayed, an explanation supported by a study assessing the impact of COVID-19 on patient-reported outcomes,^29^ showing care perceived as being less well organised. Uncertainty among patients may have resulted in adherence failures. It is also possible that, from 2020 onwards, growth in PGA prescriptions slowed because first-line treatment was changing towards SLT. Although the timeline for our analyses pre-dated the updated guideline in January 2022 recommending SLT as first-line treatment, it is likely some ophthalmic units in England were already offering SLT following publication of high-quality evidence of effectiveness.^30^

### Implications for research and practice

The present analysis adds to earlier use of large datasets in glaucoma, for example, Saunders *et al* ^31^ showing the likelihood of patients suffering visual impairment in their lifetimes being linked to visual field loss at presentation, and Kelly *et al* ^32^ observing the conversion rate of OHT to glaucoma in a retrospective examination of over 45 000 electronic glaucoma records. Our time-series analysis has shown a 1-year delay in the uptake of PGAs in more deprived areas versus national data. While lower prescribing in deprived areas may be explained by a number of factors, observing quintile two has the highest rate of PGA prescribing is perhaps counterintuitive. It is uncertain if these findings reflect prescribing being unexpectedly high in quintile two, or whether prescribing is lower than expected in quintile one. We have no evidence to suggest prevalence differs between groups, and since quintile one represents affluent areas, access to sight testing should not present a referral barrier. Arguably patients in quintiles one and two may be better informed regarding health and may seek alternative management, such as SLT or minimally invasive glaucoma procedures. Furthermore, some in quintile one may obtain private prescriptions, data which would not be captured in this study, although it would be surprising if this factor accounted for the difference of 24 prescriptions per 1000 people between quintile two and quintile one in 2021–2022.

Some evidence suggests GP practices tend to be in more affluent areas, while pharmacy achieves better levels of activity in deprived areas.^33^ Data for optometry in England is more aligned to the GP practice trend, with double the number of optometrists in the most affluent versus most deprived quintiles.^34^ In Scotland, where there is a different General Ophthalmic Services contract, distribution of optometry practices is relatively balanced across SES, with Legge *et al* ^35^ proposing differences in eye examination uptake across social strata is beyond service availability alone. The ophthalmology workstream, Getting it Right First Time,^36^ recommends an optimal glaucoma care pathway; however, inequitable distribution of eyecare has potential consequences for implementation of this approach. Integrated care boards and local authorities must ensure eyecare services are in all areas. Lower prescribing of glaucoma medications in areas of greater deprivation is an unwarranted inequality. While further studies may help establish reasons for this variation, helping development of strategies to reduce inequality, GPs working in more deprived areas can play a role in promoting the uptake of sight testing for their patients at greatest risk of glaucoma.

## Data

Study dataset can be accessed at: https://opendata.nhsbsa.net/dataset/english-prescribing-data-epd
